# Predictive value of primary gross tumor volume measured by MRI in response to neoadjuvant chemoradiotherapy in locally advanced rectal cancer

**DOI:** 10.1002/acm2.70601

**Published:** 2026-05-16

**Authors:** Junbiao Li, Yuduo Wu, Shaoping Ye, Cuiying Fan, Yanping Lin, Dianhua Yang, Xiaoyi Xia, Yajing Kang, Dehao Liu, Junxin Wu

**Affiliations:** ^1^ Department of Oncology The Third Hospital of Xiamen Xiamen China; ^2^ Medical Physics Graduate Program Duke Kunshan University Kunshan China; ^3^ School of Basic Medical Sciences Fujian Medical University Fujian China; ^4^ Department of Radiology, School of Medicine, The First Affiliated Hospital of Xiamen University Xiamen University Xiamen China; ^5^ Clinical Oncology School of Fujian Medical University Fujian Cancer Hospital Fuzhou China

**Keywords:** locally advanced rectal cancer, neoadjuvant radiotherapy, tumor volume, tumor regression grade

## Abstract

**Background:**

Neoadjuvant chemoradiotherapy (nCRT) is a standard treatment for locally advanced rectal cancer (LARC), yet individual tumor regression response varies considerably.

**Purpose:**

This study aimed to identify clinical factors linked to tumor regression grade (TRG) after nCRT in LARC and to evaluate MRI‐measured primary gross tumor volume (pGTV) as a predictor of TRG.

**Methods:**

We retrospectively analyzed 123 patients with LARC who underwent nCRT followed by total mesorectal excision (TME) and had pathologically confirmed LARC between 2014 and 2018. The pGTV was measured using MRI. Univariate and multivariate logistic regression analyses were performed to identify independent predictors of TRG. Additionally, the receiver operating characteristic (ROC) curve was employed to calculate the area under the curve (AUC) for pGTV and to determine its optimal cut‐off value in predicting TRG.

**Results:**

The study cohort comprised 52 patients in the favorable TRG group and 71 patients in the unfavorable TRG group. Both univariate and multivariate logistic regression analyses revealed that a smaller pGTV was an independent predictor of improved tumor regression (*p* < 0.001). No other clinical factors were significantly associated with TRG in the multivariate analysis. The ROC curve analysis showed that a pGTV cut‐off value of 49.25 cm^3^ yielded an AUC of 0.712 (*p* < 0.001), effectively distinguishing between the favorable and unfavorable response groups.

**Conclusions:**

MRI‐measured pGTV shows promise as a biomarker for predicting TRG after nCRT in LARC, potentially aiding pre‐treatment risk assessment. While this preliminary evidence from a retrospective, single‐center cohort requires confirmation in prospective, large‐scale studies, it provides a rationale for incorporating quantitative tumor volumetry into future research on individualized treatment strategies.

**Abbreviations**
Pgtv: Primary gross tumor volume
LARC: Locally advanced rectal cancer
Ncrt: Neoadjuvant chemoradiotherapy
TME: Total mesorectal excision
TRG: Tumor regression grade
ROC: Receiver operating characteristic
AUC: Area under curve
cCR: Clinical complete response
pCR: Pathological complete response
CEA: Carcinoembryonic antigen
EMVI: Extramural vascular invasion
CT: Computed tomography
MRI: Magnetic resonance imaging
MRF: Mesorectal fascia
CRM: Circumferential resection margin
DWI: Diffusion weighted imaging
RTOG: Radiation therapy oncology group
3D‐CRT: 3DConformalradiotherapy
IMRT: Intensity‐modulated radiation therapy
AJCC: American joint committee on cancer
HR: Hazard ratio
OR: Odds ratio
95% CI: 95% Confidence interval
DFS: Disease free survival
OS: Overall survival.

## 1 INTRODUCTION

Cancer is a significant obstacle to increasing life expectancy and a leading cause of death worldwide.^1^ The optimal treatment approach for cancer has been extensively explored, neoadjuvant treatment strategies, including neoadjuvant chemoradiotherapy (nCRT), have gained widespread acceptance.^2^, ^3^, ^4^, ^5^ For locally advanced rectal cancer (LARC), nCRT followed by total mesorectal excision (TME) surgery is now recommended as the standard treatment.^6^, ^7^ Most LARC show a reduction in tumor size and partial or complete clinical and pathologic response after nCRT. Patients who achieved clinical complete response (cCR) after nCRT may benefit from a “watch and wait” strategy^6^, potentially obviating the need of surgery. Several factors, including clinical characteristics, laboratory biomarkers, and imaging features, have been identified as having predictive value in assessing the response to nCRT.^8^, ^9^, ^10^

Smaller tumor diameter and lower clinical N stage before treatment were independent clinical predictors of achieving pathological complete response (pCR).^11^ Favorable response to nCRT was predictive of higher disease‐free survival (DFS) and overall survival (OS) in patients with rectal cancer.^12^ In a study by Gabriella et al.,^13^ the rates of pCR were 12.6%, 23%, and 31.1% (*p* < 0.001) for patients with an interval between radiotherapy and TME surgery of within 6 weeks, 7–12 weeks, and over 13 weeks, respectively. Tumor Regression Grade (TRG) is also a novel independent predictor of survival prognosis. Patients with a good TRG exhibited a more favorable prognosis, lower local recurrence rates, fewer distant metastases, and longer OS.^14^ Therefore, early and accurate prediction of LARC tumor regression response to nCRT is crucial for optimizing treatment strategies.^15^ However, limited research has focused on the impact of primary gross tumor volume (pGTV) on the response to nCRT. The difficulty in contouring the volume of rectal cancer and the substantial variation in volume measurements based on different imaging techniques have led to discrepancies between studies regarding the pGTV volume cut‐off for predicting nCRT response.^16^, ^17^

Magnetic resonance imaging (MRI) is extensively employed as a pivotal imaging modality for the staging of rectal cancer and the assessment of tumor regression following nCRT.^18^, ^19^, ^20^ A prospective work^21^ revealed a robust correlation between the tumor extent evaluated by MRI and histopathological examination. MRI‐based evaluations offered the advantage of a more precise delineation of the extent of rectal cancer infiltration and the determination of pGTV. Moreover, our measurement of pGTV utilized pre‐treatment high‐resolution MRI, maintaining direct correspondence with standard diagnostic and staging protocols. This congruence facilitated its straightforward integration and broader adoption in clinical settings. Therefore, Utilizing MRI to obtain parameters such as pGTV in LARC, in conjunction with additional data including patient gender, age, and serum carcinoembryonic antigen (CEA) levels, allows for the evaluation of the predictive value of these factors for treatment response.

## 2 MATERIALS AND METHODS

### 2.1 Patients

Patients who visited the Radiotherapy Center between February 2014 and May 2018 were retrospectively analyzed. We carried out all procedures in accordance with the Helsinki Declaration. This survey was conducted in an anonymous form. All participants signed the informed consent form and indicated their agreement by selecting ‘Agree’ on it. This study included 123 individuals with LARC who received nCRT. To be eligible for inclusion, patients had to meet the following criteria: (1) they underwent a complete abdominopelvic MRI before treatment, were diagnosed with clinical stage T3, 4, and/or N+ LARC, and received neoadjuvant long‐course radiotherapy followed by radical TME surgery; (2) they provided informed consent and voluntarily participated in the study. Exclusion criteria were: (1) interruption of treatment; (2) a history of other tumors or hereditary colorectal cancer at the time of diagnosis.

### 2.2 nCRT

The Radiation Therapy Oncology Group (RTOG) consensus^22^ was utilized to ascertain the patients' tumor volumes. The radiation dose was prescribed at 45–50 Gy (1.8‐2 Gy per fraction, delivered 5 times per week) using either 3D conformal radiotherapy (3D‐CRT) or intensity‐modulated radiation therapy (IMRT) techniques. Concurrently, 5‐Fluorouracil (5‐FU)‐based neoadjuvant chemotherapy was administered. Radical TME surgery was performed 4–12 weeks following the completion of nCRT.

### 2.3 Variables selection

**FIGURE 1:**
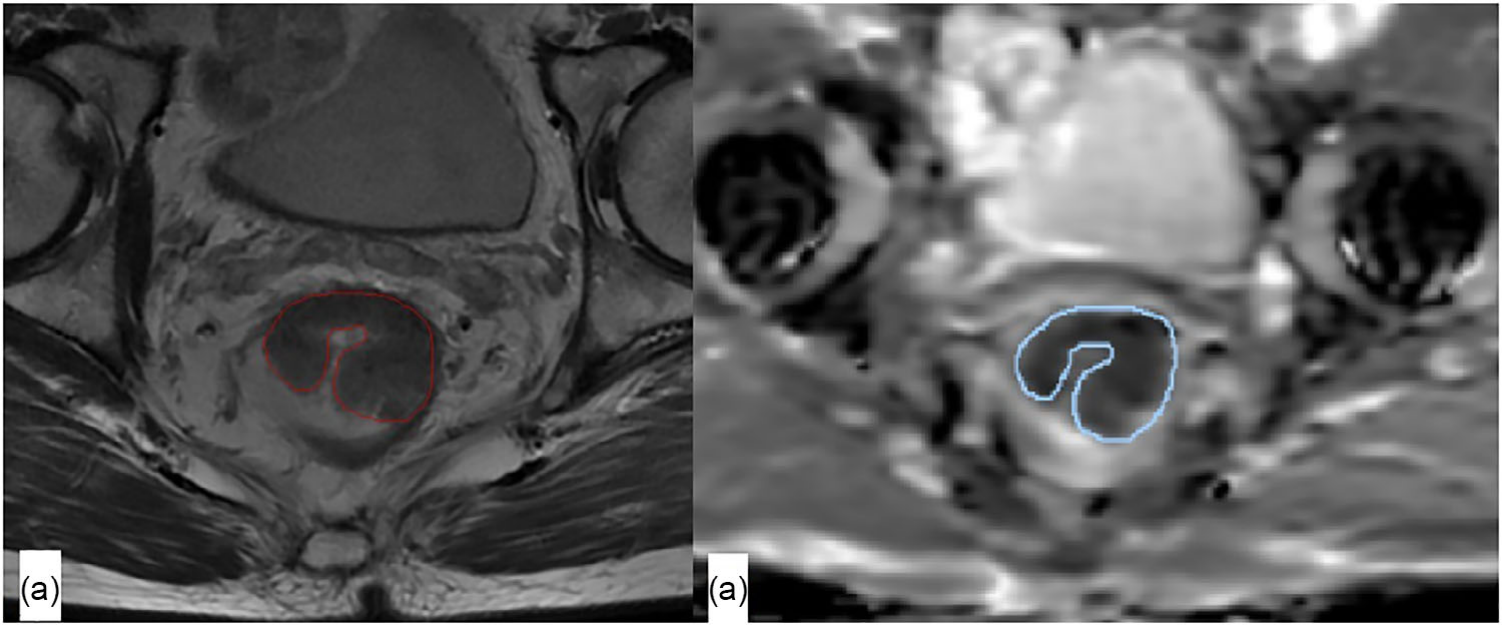
Imaging sequences and tumor volume calculation. (a) FOV and axial T2WI sequences; (b) Axial apparent diffusion coefficient (ADC) sequence. The left anterior half of the intestinal wall was thickened. The tumor infiltrated the proper muscle layer and involved the mesorectum, with evidence of extramural venous invasion (EMVI). The regions of interest (ROIs) were delineated on a para‐axial high‐resolution T2‐weighted sequence and adjusted with reference to diffusion‐weighted imaging (DWI)/ADC sequences. The tumor boundaries were traced layer by layer, and the tumor volume (in cm^3^) was automatically calculated using TPS software.

All patients underwent conventional axial T2‐weighted pre‐saturated fat suppression imaging, T1‐weighted imaging and diffusion‐weighted imaging scans (with a layer thickness of 5mm and a layer spacing of 0.5mm), as well as nonfat suppression high‐resolution T2WI (axial, sagittal, coronal) scans (with a layer thickness of 3mm and a layer spacing of 0.5mm). The MRI images were transferred to the Monaco treatment planning system (TPS) for processing. Following image pre‐processing on the TPS, the pGTV was delineated independently by two abdominal imaging specialists and one radiation oncologist, each blinded to the others' assessments. The pGTV was then automatically calculated by the TPS software. In cases of disagreement, a final consensus contour was reached through discussion (Figure 1). Finally, the variables selected for analysis were as follows: (1) general and staging information (gender, age, clinical T and N stages); (2) imaging and laboratory data (pGTV, tumor circumference, extramural vascular invasion (EMVI), tumor distance from the anal verge, peritoneal fold involvement, and serum CEA level before nCRT); and (3) postoperative pathological results (TRG).

### 2.4 Statistical analysis

In the descriptive analysis, the one‐sample Kolmogorov‐Smirnov (K‐S) test was used to determine whether the continuous variables were normally distributed. For normally distributed variables, the mean and standard deviation were reported; otherwise, the median (with quartiles) was used. The frequency (%) was utilized for categorical variables. Pathological response was classified into two groups based on the AJCC TRG system: the good regression group (TRG 0–1) and the poor regression group (TRG 2–3). To test the significance of differences between our two distinct TRG groups for continuous variables, the Mann‐Whitney *U*‐test or the two independent samples *t*‐test were employed, depending on the data distribution. For categorical variables, the chi‐square (*χ* ^2^) test or Fisher's exact test was used. Univariate and multivariate logistic regression analyses were performed to identify the variables of interest related to TRG. Receiver operating characteristic (ROC) curves were used to calculate the optimal cutoff value of pGTV for predicting TRG. The Youden Index was used to determine the optimal cutoff value for pGTV, maximizing overall diagnostic accuracy. The statistical tests were conducted using RStudio (version 4.2.2), including the Table 1 and pROC packages. A *P*‐value less than 0.05 was considered statistically significant.

**TABLE 1:** Baseline clinicopathological features in patients with LARC.

**Variables**	**Overall (*N* = 123)**
Age(years) mean (±SD)	54 (±10.9)
Sex	
Male	84 (68.3%)
Female	39 (31.7%)
CEA(U/ml) median (Min, Max)	3.55 (0.100, 161)
Missing	5 (4.1%)
Distance from the anal verge(cm) median (Min, Max)	5.0 (0.5, 15.0)
Missing	1 (0.8%)
Peritoneal fold involvement	
Negative	52 (42.3%)
Positive	71 (57.7%)
Tumor circumference median (Min, Max)	0.75(0.25, 1)
Missing	5 (4.1%)
pGTV(cm3) median (Min, Max)	38.4 (13.0, 301)
EMVI	
Negative	27 (22.0%)
Positive	88 (71.5%)
Missing	8 (6.5%)
cT stage	
cT2	2 (1.6%)
cT3‐4	121 (98.4%)
cN stage	
cN0	16 (13.0%)
cN1‐2	107 (87.0%)
Radiotherapy type	
3D‐CRT	20 (16.3%)
IMRT	103 (83.7%)
Surgery interval(weeks) median (Min, Max)	8 (3, 14)
Pathological stage	
No pCR	105 (85.4%)
pCR	18 (14.6%)
TRG	
0‐1	52 (42.3%)
2‐3	71 (57.7%)
Differentiation	
Poor	3(2.4%)
Moderate‐well	120 (97.6%)
CRM	
Negative	51 (41.5%)
Positive	65 (52.8%)
Missing	7 (5.7%)
LVI;	
Negative	102 (82.9%)
Positive	21 (17.1%)
PNI	
Negative	109 (88.6%)
Positive	14 (11.4%)
ypT stage	
T0‐2	44 (35.8%)
T3‐4	79 (64.2%)
ypN stage	
N0	77 (62.6%)
N1‐2	46 (37.4%)

Abbreviation: CEA, carcinoembryonic antigen; pGTV, primary gross tumour volume; TRG, tumor regression grade; pCR, pathological complete response; 3D‐CRT, 3D conformal radiotherapy; IMRT, intensity‐modulated radiation therapy.

## 3 RESULTS

### 3.1 Clinical characteristics of the small study cohort

This study enrolled 123 patients diagnosed with LARC, characterized by a median age of 54 years and a male predominance (68%). The median concentration of CEA was 3.55 U/mL. The median distance from the anal verge to the tumor was 5 cm, with 75% of the tumor circumference involved. The median pGTV was 38.4 cm^3^ (ranging from 13 to 301 cm^3^). A high proportion of patients (71.5%) exhibited positive EMVI. All patients received nCRT, with IMRT used in 83.7% of cases and 3D‐CRT in 16.3%. The TME was carried out 8 weeks post‐nCRT (ranging from 3 to 14 weeks). Postoperative pathological examination confirmed adenocarcinoma in all patients, with the majority (97.6%) demonstrating moderate or well differentiation. A pCR was achieved in 18 patients (14.6%). Poor TRG was observed in 71 patients (57.7%), whereas a good TRG was noted in 52 patients (42.3%). The positivity rates for circumferential resection margin (CRM), lymphovascular invasion (LVI), and perineural invasion (PNI) were 52.8%, 17.1%, and 11.4%, respectively. In comparison, fewer patients had pathologic stages of T3‐4 (98.4%) and N1‐2 (87.0%) than the corresponding clinical stages of T3‐4 (64.2%) and N1‐2 (37.4%) (Table 1).

### 3.2 Correlation between pGTV and TRG

Further analysis indicated that only the pGTV (*P* < 0.001) among the clinical characteristics of the patients (Table 2) was statistically significantly different between the two TRG groups. Other factors, such as age, sex, CEA level, distance from the anal verge, involvement of the peritoneal fold, tumor circumference, etc., were not significantly different. Additionally, univariate analysis was performed to identify clinical factors associated with TRG. Multivariate analysis included factors whose *p*‐values in the univariate analysis were less than 0.2. Only pGTV (*p* < 0.001) was found to be significantly associated with TRG and served as an independent predictor (Table 3).

**TABLE 2:** Clinical traits and TRG.

**Variables**	**TRG 0‐1** **(*N* = 52)**	**TRG 2‐3** **(*N* = 71)**	***P* value**
Age (year)	54.4 (±12.3)	53.7 (±9.82)	0.758
Gender			1
male	36 (69.2%)	48 (67.6%)	
female	16 (30.8%)	23 (32.4%)	
CEA (U/mL)	3.45 (0.1, 72.8)	3.55 (0.100, 161)	0.9
Missing	2 (3.8%)	3 (4.2%)	
Distance from the anal verge (cm)	5.00 (0.5, 11.0)	5.00 (1.90, 15.0)	0.272
Missing		1 (1.4%)	
Peritoneal fold involvement			0.353
Negative	25 (48.1%)	27 (38.0%)	
Positive	27 (51.9%)	44 (62.0%)	
Tumor circumference	0.75 (0.25, 1)	0.75 (0.5, 1)	0.205
Missing	1 (1.9%)	4 (5.6%)	
pGTV	31.5 (13.0, 74.0)	43.6 (23.0, 301)	**< 0.001**
EMVI			0.499
Negative	14 (26.9%)	13 (18.3%)	
Positive	37 (71.2%)	51 (71.8%)	
Missing	1 (1.9%)	7 (9.9%)	
cT stage			1
cT2	1 (1.9%)	1 (1.4%)	
cT3‐4	51 (98.1%)	70 (98.6%)	
cN stage			0.346
cN0	9 (17.3%)	7 (9.9%)	
cN1‐2	43 (82.7%)	64 (90.1%)	
Radiotherapy type			0.982
3D‐CRT	9 (17.3%)	11 (15.5%)	
IMRT	43 (82.7%)	60 (84.5%)	
Surgery interval (week)	7.5 (3, 14)	8 (3, 13)	0.551

Abbreviation: TRG, tumor regression grade; CEA, carcinoembryonic antigen; pGTV, primary gross tumour volume; 3D‐CRT, 3D conformal radiotherapy; IMRT, intensity‐modulated radiation therapy.

**TABLE 3:** Independent predictors of TRG.

		Univariate		Multivariate	
Variable		OR (95%CI)	*P* value	OR (95%CI)	P value
Gender	Male	Ref	0.848		
	Female	1.078(0.501–2.355)	0.848		
CEA		1.003(0.989–1.019)	0.695		
Age		0.995(0.962–1.028)	0.747		
pGTV		1.042(1.020–1.070)	<0.001	1.043(1.015–1.077)	**<0.001**
Distance from the anal verge		1.128(0.947–1.362)	0.190	1.128(0.932–1.388)	0.222
Peritoneal fold involvement	Negative	Ref	0.265		
	Positive	1.509(0.731–3.131)	0.266		
Tunor circumference		4.584(0.597–37.973)	0.148	1.339(0.133–13.694)	0.803
Tumor length		1.154(0.988–1.364)	0.078	0.960(0.786– 1.171)	0.681
EMVI	Negative	Ref	0.371		
	Positive	1.484(0.623–3.563)	0.371		
cT stage	cT2	Ref	0.825		
	cT3‐4	1.373(0.053–35.255)	0.824		
cN stage	cN0	Ref	0.228		
	cN1‐2	1.914(0.664–5.727)	0.230		

Abbreviation: CI, confidence interval; pGTV, primary gross tumour volume; OR, odds ratio.

### 3.3 Optimal cut‐off value of pGTV for predicting TRG

**FIGURE 2:**
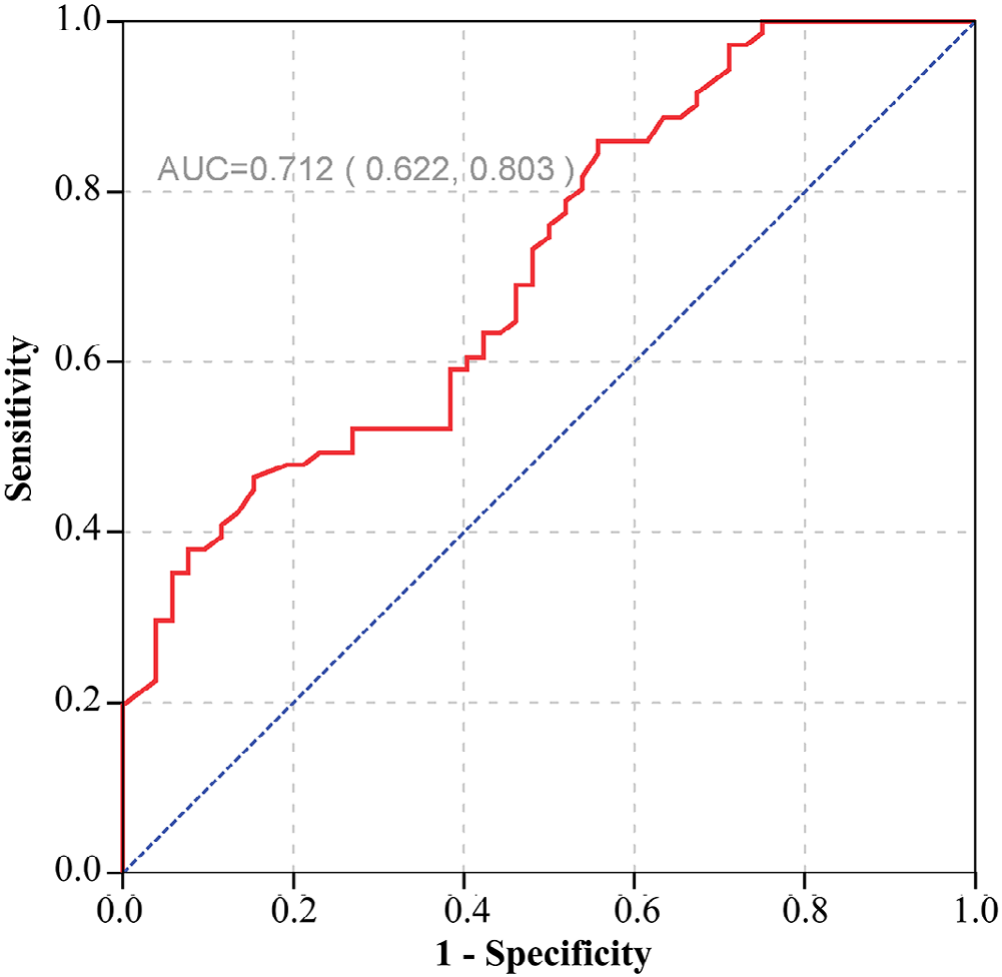
Area under the curve (AUC) for predicting the grade of tumor regression based on a cut‐off value determined by the pGTV.

The optimal cut‐off value for pGTV, as determined through ROC curve analysis, was 49.25 cm^3^. This cut‐off value maximized the Youden Index, a statistical measure which combines sensitivity and specificity to assess a diagnostic test's overall performance. At this cut‐off, the sensitivity was 46.5% (95% CI: 0.347–0.589), while the specificity was 84.6% (95% CI: 0.722–0.928). Furthermore, the AUC for predicting TRG using pGTV was 0.712 (95% CI: 0.622–0.803) (Figure 2), demonstrating that pGTV has good ability to distinguish between patients with different TRG categories. These findings highlighted the potential clinical utility of pGTV as a predictive biomarker for TRG in patients undergoing treatment for LARC.

## 4 DISCUSSIONS

The eighth edition of the American Joint Committee on Cancer (AJCC) classification system for rectal cancer *T*‐stage incorporates the depth of tumor infiltration into the intestinal wall as a critical factor.^23^ Nonetheless, it is imperative to acknowledge that substantial variability in tumor volume may exist even among tumors assigned to the same *T*‐stage. Neglecting this inherent heterogeneity can jeopardize the validity and reliability of research findings.^24^ Our study identified pGTV as an independent predictor of TRG in patients with LARC undergoing nCRT. Specifically, a pGTV of less than 49.25 cm^3^ prior to nCRT was associated with a favorable response.

Previous studies supported the biological plausibility of this association. Larger tumor volumes were often linked to increased radioresistance, potentially due to factors such as hypoxia within the tumor mass.^25^ Several investigations have explored pGTV as a predictor, though methodologies and results vary. Liu et al.^16^, utilizing radiotherapy planning CT, reported a significant correlation and a cut‐off value of 71.06 cm^3^ for predicting post‐nCRT regression. Lutsyk et al.^17^, using preoperative MRI, found a pGTV < 39.5 cm^3^ to be an independent predictor of pCR. While consistent with our findings in direction, the differing cut‐off values highlight the impact of measurement techniques (CT vs. MRI), study cohort characteristics, and the defined endpoint (pCR vs. favorable TRG). Notably, O'Neill et al.^26^ demonstrated that, in 3D‐CRT, pre‐treatment estimations of rectal tumor volume and length were significantly larger on planning CT compared to MRI.

However, our study extends beyond mere replication by offering several novel contributions that strengthen its clinical value. First, unlike Liu et al.^16^ who utilized planning CT, our method validates the predictive capability of pGTV derived from routine diagnostic MRI. Given the superior soft tissue resolution of MRI for delineating rectal tumor boundaries, this approach ensures greater accuracy and reproducibility, and it enhances clinical translatability as diagnostic MRI is universally performed without the need for additional imaging. Second, while Lutsyk et al.^17^ dichotomized response into pCR and non‐pCR, our study employed a clinically relevant grouping of favorable (TRG 0–1) versus poor (TRG 2–3) response. This broader definition captures a wider spectrum of patients who might benefit from organ‐preserving strategies, reflecting a distinct clinical priority. Finally, the specific threshold of 49.25 cm^3^ identified in our institutional context adds a quantitative reference for centers with similar patient profiles and treatment regimens. Collectively, these distinctions underscore that our approach offers complementary predictive insights beyond those previously reported.

Beyond its role in predicting nCRT response, the prognostic value of pGTV extends to longer‐term oncologic outcomes, both in rectal cancer and other malignancies. In terms of prognosis, Jiang et al.^27^ conducted a review involving 270 patients with pathologically confirmed early‐stage rectal cancer (T1/2, N0) and found that pGTV was an independent predictor of DFS and local recurrence‐free survival (LRFS), although no significant difference was observed in OS. This finding aligns with observations in other cancer types, where pGTV has demonstrated a strong association with prognosis. For instance, Fen et al.^28^ investigated 159 patients with stage T3‐4 nasopharyngeal carcinoma and reported that pGTV, as well as the volume of tumor invasion into adjacent structures such as the skull base, were significantly correlated with local failure. Specifically, a larger invasion volume (≥22 mL) was identified as an independent predictor of unfavorable local control. Similarly, Icht et al.^29^ observed that a smaller pGTV was associated with higher response rate (RR), progression‐free survival (PFS), and OS in patients with advanced non‐small cell lung cancer (NSCLC) treated with immune checkpoint inhibitors (ICIs).

In contrast to the work of Yang^30^ and Zhong^31^, which focused on predicting pCR (TRG 0 vs. 1–3), our study differentiates between a good response group (TRG 0–1) and a poor response group (TRG 2–3). This approach of consolidating TRG 0–1 into a single favorable category, despite possibly reducing specificity due to increased group heterogeneity, is intended to enhance sensitivity. This trade‐off reflects our clinical priority: to capture as many potential good responders as possible to facilitate more aggressive conservative management, even at the cost of some false‐positive classifications.

Due to its relatively low soft tissue resolution, CT imaging has difficulty in precisely delineating tumor boundaries from normal intestinal walls or inflammatory tissues, often leading to overestimation or underestimation of tumor volume. In contrast, MRI provides exceptional anatomic detail owing to its high soft tissue resolution. For the accurate measurement of rectal cancer tumor volume, MRI is comprehensively superior to CT imaging in terms of accuracy, reproducibility, and clinical relevance. Unlike the study by Liu et al.^16^ which was based on the CT measurement volume of the radiotherapy plan, our method directly validates the predictive capability of tumor volume as seen on routine diagnostic MRI, making it more readily translatable into clinical practice. The research by Lutsyk et al.^17^ dichotomized posttreatment pathological response into pCR and non‐pCR groups, reporting a cut‐off value of 39.5 cm^3^, which differs from our result of 49.25 cm^3^. This discrepancy may be attributed to variations in baseline population characteristics, the interval to surgery, or differences in the predefined pathological response groups across studies. It also underscores that the predictive threshold of tumor volume may vary depending on the population, surgery interval, and treatment regimen. Therefore, our study is not a mere replication but provides a specific, internally validated quantitative reference for centers with similar clinical contexts.

As a continuous variable, pGTV potentially encapsulates more comprehensive information regarding tumor burden compared to categorical variables (e.g., cT stage, EMVI). Given that tumor burden constitutes a fundamental biological determinant of chemoradiotherapy response, pGTV assumed a dominant role in the multivariable analysis. In our study cohort, pre‐treatment MRI‐measured pGTV emerged as a strong predictor, independent of other conventional clinicopathological factors. This finding implies that incorporating quantitative volumetric assessment can yield additional and clinically valuable prognostic information beyond the existing clinical staging system.

The identification of a pGTV cut‐off value carries direct clinical implications for risk‐adapted management in LARC. Patients with a large pGTV (≥ 49.25 cm^3^) are at higher risk of poor regression after nCRT. For this subset of patients, clinicians may consider treatment intensification (e.g., consolidation chemotherapy), more extensive surgical planning, or enhanced surveillance. Conversely, for patients with a small pGTV (< 49.25 cm^3^), especially when combined with favorable post‐nCRT clinical and MRI findings, the likelihood of achieving a good response is increased. This quantitative metric can bolster confidence in considering organ‐preserving approaches, such as a ‘watch‐and‐wait’ strategy or local excision. Integration of baseline pGTV with post‐nCRT restaging MRI can further refine surgical planning, aiding in decisions regarding the extent of resection and the appropriateness of sphincter‐preserving techniques.

pGTV constitutes a macroscopic morphological measure that does not account for intratumoral microscopic heterogeneity. Addressing this constitutes a central avenue for future work: the synergistic integration of anatomical volumetrics with functional/quantitative imaging biomarkers (e.g., ADC) to construct next‐generation predictive models, thus steering LARC treatment strategies toward a more sophisticated era of precision medicine. Future prospective studies should integrate multiparametric MRI (including IVIM or DCE) to investigate whether ‘functional volume’ delineation based on biological characteristics offers superior predictive performance compared to traditional anatomical volume.

The performance characteristics of the identified pGTV cut‐off are crucial for its clinical interpretation. The high specificity (84.6%) means that a pGTV < 49.25 cm^3^ reliably identifies patients likely to have a good response, making it a valuable supportive criterion for conservative management. Conversely, the moderate sensitivity (46.5%) indicates that this metric alone should not be used to exclude patients from such strategies, as many good responders would be missed. Therefore, pGTV is best positioned as a key component within a multimodal decision‐making framework rather than a standalone triage tool.

It must be acknowledged that this study has several limitations. First, its retrospective, single‐center design carries inherent risks of selection and information bias. Second, the modest sample size limited our ability to perform robust subgroup analyses or external validation. These constraints necessitate that our findings be considered preliminary and underscore the imperative for validation in larger, prospective, and preferably multi‐institutional cohorts. Looking forward, pGTV represents a macroscopic anatomical parameter. Future research should focus on integrating it with functional imaging biomarkers and molecular profiles to build more comprehensive predictive models. This synergistic approach represents a promising pathway toward advancing precision medicine in LARC.

In conclusion, pre‐treatment MRI‐measured pGTV is an independent predictor of pathological response to nCRT in LARC. It provides a quantifiable, readily available metric that can enhance pre‐therapeutic risk stratification and inform personalized treatment planning, complementing existing clinical assessments.

## AUTHOR CONTRIBUTIONS

All authors listed have made a substantial, direct and intellectual contribution to the work, and approved it for publication. **Junbiao Li** designed and wrote the main manuscript text. **Junxin Wu** designed the main study. **Yuduo Wu** and **yanping Lin** analyzed and counted the data. **Shaoping Ye** prepared figures. All authors reviewed the manuscript. All authors read and approved the final manuscript.

## FUNDING

This research was funded by the Fujian Clinical Research Center for Radiation and Therapy of Digestive, Respiratory and Genitourinary Malignancies (2021Y2014).

## CONFLICT OF INTEREST STATEMENT

The authors declare that they have no competing interests.

## ETHICS STATEMENT

Patients who visited the Radiotherapy Center at Fujian Cancer Hospital between February 2014 and May 2018 were retrospectively analyzed. This study was conducted under the ethical guidelines of the Helsinki Declaration and approval by the Ethics Committee of Fujian Cancer Hospital (K2021‐050‐01). We carried out all procedures in accordance with the Helsinki Declaration. This survey was conducted in an anonymous form.

## CONSENT

All participants signed the informed consent form and indicated their agreement by selecting ‘Agree’ on it.

## Data Availability

All data generated or analyzed during this study are included in this article. Further enquiries can be directed to the corresponding author.
